# Prevalence of rapid response systems in small hospitals: A questionnaire survey

**DOI:** 10.1097/MD.0000000000026261

**Published:** 2021-06-11

**Authors:** Koji Hosokawa, Hiroki Kamada, Kohei Ota, Satoshi Yamaga, Junki Ishii, Nobuaki Shime

**Affiliations:** a Department of Emergency and Critical Care Medicine, Graduate School of Biomedical and Health Sciences, Hiroshima University, Hiroshima, 1-2-3 Kasumi, Minami-ku, Hiroshima; b Department of Anesthesiology and Reanimatology, Faculty of Medical Sciences, University of Fukui, 23-3 Eiheijicho, Yoshidagun, Fukui; c Department of Medicine, Hiroshima University, Hiroshima, 1-2-3 Kasumi, Minami-ku, Hiroshima, Japan.

**Keywords:** epidemiology, in-hospital cardiac arrest, medical safety management, rapid response system

## Abstract

The rapid response system (RRS) was introduced for early stage intervention in patients with deteriorating clinical conditions. Responses to unexpected in-hospital patient emergencies varied among hospitals. This study was conducted to understand the prevalence of RRS in smaller hospitals and to identify the need for improvements in the responses to in-hospital emergencies.

A questionnaire survey of 971 acute-care hospitals in western Japan was conducted from May to June 2019 on types of in-hospital emergency response for patients in cardiac arrest (e.g., medical emergency teams [METs]), before obvious deterioration (e.g., rapid response teams [RRTs]), and areas for improvement.

We received 149 responses, including those from 56 smaller hospitals (≤200 beds), which provided fewer responses than other hospitals. Response systems for cardiac arrest were used for at least a limited number of hours in 129 hospitals (87%). The absence of RRS was significantly more frequent in smaller hospitals than in larger hospitals (13/56, 23% vs 1/60, 2%; *P* < .01). METs and RRTs operated in 17 (11%) and 15 (10%) hospitals, respectively, and the operation rate for RRTs was significantly lower in smaller hospitals than in larger hospitals (1/56, 2% vs 12/60, 20%; *P* < .01). Respondents identified the need for education and more medical staff and supervisors; data collection or involvement of the medical safety management sector was ranked low.

The prevalence of RRS or predetermined responses before obvious patient deterioration was ≤10% in small hospitals. Specific education and appointment of supervisors could support RRS in small hospitals.

## 1 Introduction

In-hospital management of cardiac arrest is of clinical interest.^[1]^ Traditionally, a code call is an emergency response system for patients with cardiac arrest or close to cardiac arrest.^[2]^ As some physiological derangements are frequently observable several hours before a cardiac arrest,^[3]^ the rapid response system (RRS) was developed for early stage identification of patients at risk for rapid clinical decline.^[4]^ The intervention of the rapid response team (RRT) before a catastrophic event can reduce in-hospital mortality rates.^[5,6]^ Together with the medical emergency team (MET), which initiates advanced life support during clinical status deterioration, RRTs/METs are recommended for the implementation of acute-care preparedness in hospitals.^[7,8]^

In alignment with global trends, the introduction of the RRS is reflected in the Japanese literature around 2010,^[9,10]^ followed by the launching of several action goals, including the RRS, by the Japanese Coalition for Patient Safety.^[11]^ The Japanese Intensive Care Society and others published uniform terminology with regard to the RRS.^[12]^ The Ministry of Health, Labour, and Welfare incorporated new policies for the detailed evaluation of advanced emergency and critical care centers.^[13]^

Despite its importance, the prevalence of the RRS in Japan is not well known, although it is possibly influenced by the hospital care type and staff capacity.^[14,15]^ Recently, Naito et al^[16]^ published a descriptive summary from 35 institutes that registered activity data of their RRT/MET to an In-Hospital Emergency Committee in Japan (IHEC-J).^[17,18]^ This depicts the status of large acute-care hospitals. An evaluation that includes smaller hospitals is necessary, given that a considerable number of smaller hospitals are involved in acute-care provision in Japan.

This study was undertaken to identify the prevalence and types of response systems for in-hospital emergencies depending on hospital volume. We investigated respondents’ perspectives on the need for improvement in responses to in-hospital emergencies.

## 2 Methods

We conducted a questionnaire survey of hospitals with ≥75 beds in 17 prefectures in the western districts of Japan, including at least 10 beds for acute care. We used the designated hospital databases of the Chugoku-Shikoku (https://kouseikyoku.mhlw.go.jp/kyushu/) and Kyushu (https://kouseikyoku.mhlw.go.jp/kyushu/) Regional Bureau of Health and Welfare, and the number of beds shown on the website (https://www.mhlw.go.jp/stf/seisakunitsuite/bunya/0000055891.html). We sent 971 invitation letters to hospital administrators requesting their participation in a questionnaire survey in May (nine prefectures in Chugoku-Shikoku) and June (eight prefectures in Kyushu) 2019. Respondents were selected by each hospital without any incentive to respond. The response deadline cut-off was set at 21 days from the date of the letter.

The survey questionnaire was developed in Google Forms and included the hospital identification code, respondent's department, data collection at each hospital, participation of data registry to the IHEC-J, type of activity for patients in clinical crisis or in cardiac arrest, activity undertaken before obvious clinical status deterioration, and need for improvement in responses to in-hospital emergencies (Table S1, Supplemental Digital Content).

One section of questions that were response to cardiac arrest included a staff call, a preassigned doctor's call, calling for public emergency services, and MET. Another section of RRS included RRT, critical care outreach team (CCOT), etc. The staff call was a code called to assemble nonspecific medical staff to the site of the emergency (e.g., Code Blue). The preassigned doctor's call was the call for the arrival of specific medical staff, including a medical doctor, in an emergency situation. The MET, RRT, and CCOT were described in Japanese, as defined by the Joint Committee on Rapid Response System of the Japanese Society of Intensive Care Medicine and the Japanese Society for Emergency Medicine.^[12]^ The MET/RRT may act in both situations, although the MET and RRT were indicated in the first and second sections of the questionnaire, respectively, and blank or multiple answers were allowed. To identify hospitals, the respondents included hospital codes in the questionnaire.

We collated and analyzed data to compare the differences between smaller hospitals (≤200 beds) and other hospitals. Statistical analysis was performed using Fisher exact test on the JMP 13 (SAS Institute Inc., Cary, NC); *P* < .05, considered statistically significant.

The need for ethics approval was waived by the ethics committee because this questionnaire survey did not involve research on patients.

## 3 Results

We received an overall response from 149 hospitals (response rate 15%), with 56 small hospitals (≤200 beds) from which there was a lower response rate than that from larger hospitals (9% vs 17%; Table 1). Sixty large and 10 university hospitals were included. Respondents belonged to various departments in each hospital, with those from the medical safety management sectors constituting 18% of the total responders. The rate of response from non-doctors (i.e., nurses or administrative officers) was higher in smaller hospitals (Table 1). Twenty-three hospitals (15%) collected response data of hospital emergencies and 7 hospitals (5%) registered their data in the IHEC-J registry, and most were larger hospitals.

**Table 1:** Summary of respondents.

		Number of beds		
		≤200	≥201	Unidentified
Request for the questionnaire	971	623	348	
Response (% of requests)	149 (15%)	56 (9%)	60 (17%)	33
Respondents				
Section of medical safety management	27 (18%)	9 (16%)	9 (15%)	9 (27%)
Doctors in emergency medicine, anaesthesiology and others	57 (38%)	14 (25%)	37 (62%)	6 (18%)
Nurses in emergency, administrative and others section	37 (25%)	17 (30%)	9 (15%)	11 (33%)
Officers in administrative office	20 (13%)	11 (20%)	3 (5%)	6 (18%)
Others or unspecified	8 (5%)	5 (9%)	2 (3%)	1 (3%)
Data collection	23 (15%)	2 (4%)	13 (22%)	8 (24%)
Data registration to IHEC-J registry	7 (5%)	0 (0%)	5 (8%)	2 (6%)

Values were shown in number (percentage of responses in each category). IHEC-J = In-Hospital Emergency Committee in Japan.

### 3.1 Code blue and MET for cardiac arrest

Responses for patients with unexpected clinical status decline or cardiac arrest were predetermined in 129 hospitals (87%; Table 2). A full-time system was available for 82 hospitals (55%). Twenty hospitals (13%) did not have rules or defined activities for the management of patients who unexpectedly deteriorated or experienced cardiac arrest. The rate of absence of response in small hospitals was significantly higher than that in larger hospitals (13 of 56, 23% vs 1 of 60, 2%; *P* < .01).

**Table 2:** Prevalence of response activities for in-hospital emergency and RRS.

		Number of beds		
	Total response (n = 149)	≤200 (n = 56)	≥201 (n = 60)	Unidentified (n = 33)
Code blue and MET for cardiac arrest				
Any system, full time	82 (55%)	19 (34%)	44 (73%)	19 (58%)
MET, full time	12 (8%)	1 (2%)	9 (15%)	2 (6%)
Any system, limited hours	47 (32%)	24 (43%)	15 (25%)	8 (24%)
MET, limited hours	5 (3%)	0 (0%)	4 (7%)	1 (3%)
No services	20 (13%)	13 (23%)	1 (2%)	6 (18%)
RRS using RRT or others				
Any system, full time	7 (5%)	1 (2%)	5 (8%)	1 (3%)
RRT, full time	7 (5%)	1 (2%)	5 (8%)	1 (3%)
Any system, limited hours	10 (7%)	1 (2%)	8 (13%)	1 (3%)
RRT, limited hours	8 (5%)	0 (0%)	7 (12%)	1 (3%)
No services	132 (89%)	54 (96%)	47 (78%)	31 (94%)

MET = medical emergency team, RRS = rapid response system, RRT = rapid response team.

A staff call code was used in 117 hospitals (79%), covering both inpatients and outpatients and incidents in the hospital building in 88 hospitals (75%), and incidents at sites around the hospital building in 25 hospitals (21%). Seventy hospitals (60%) were operated for 24 hours. The rate of operation of staff calls in smaller hospitals was lower than that in larger hospitals (39 of 56, 70% vs 52 of 60, 87%; *P* = .04). All 7 hospitals that registered their data in the IHEC-J registry operated staff calls.

A preassigned doctor's call was operational in 48 hospitals (32%). The response staff included emergency doctors (21, 44%), anesthesiologists (19, 40%), and intensivists (9, 19%). Moreover, protocols for calling public emergency services (#119 in Japan) were used in 19 hospitals.

The METs were operational in 17 hospitals (11%). The median number of beds in these hospitals was 598 (interquartile range, 324–663). Four university hospitals operated METs. Data were collected from 10 hospitals and registered in the IHEC-J registry of 4 hospitals.

### 3.2 RRS using RRT and others

Response activity before patient deterioration or cardiac arrest was operational in 17 hospitals (11%), whereas 15 hospitals (10%) had RRTs. The operation rate of RRTs was significantly lower than that in smaller hospitals (≤200 beds) than in larger hospitals (1 of 56 vs 12 of 60, *P* < .01). The RRTs included emergency doctors, anesthesiologists, or intensivists in 10 hospitals. CCOTs were operational in 5 hospitals and all operated RRTs. Two hospitals operated both RRTs and CCOTs, which were mainly managed by nurses. Other RRS activities included nurses’ rounds and rules for referral and transport of patients at risk to higher-level hospitals.

### 3.3 Need for improvement

The need for improvement in response activity for in-hospital emergencies was diverse (Table 3). The high-ranked needs of respondents who were less satisfied with activities to in-hospital emergency were related to education of knowledge, the number of staff, positive attitude of staff, presence of supervisors, and regulations related to RRS. However, the involvement of a medical safety management team or data collection was ranked low.

**Table 3:** Satisfaction and areas for improvement of RRS.

	Total response (n = 149)
Grade of satisfaction	0.6 [0.4–0.6]
Category of satisfaction	
0–0.4	62 (42%)
0.5–1	85 (57%)
Needs of respondents in low satisfaction grade (0–0.4) (n = 62)	
Education of knowledge/improvement of protocol	57 (92%)
Number of staff/positive attitudes	52 (84%)
Multidisciplinary approach	50 (81%)
Presence of leaders or doctors	48 (77%)
Regulation	42 (68%)
Less frequent needs of respondents	
Increase of equipment	79 (53%)
Registration of data	49 (33%)
Involvement of medical safety management sector	43 (29%)

The grade of satisfaction ranged from 0 to 1. Values were shown in median [interquartile range] or number (%). RRS = rapid response system.

## 4 Discussion

The present questionnaire survey revealed that any type of code call for unexpected cardiac arrest was widespread in larger acute-care hospitals. However, in smaller hospitals, the prevalence of MET or RRS activation before obvious patient deterioration was remarkably low. The inclusion of smaller hospitals is a new paradigm in the research on hospital emergency response systems, since the reports regarding RRS in hospitals were only selected institutes in the United State,^[19,20]^ the United Kingdom,^[21]^ Australia,^[22]^ New Zealand,^[23]^ and Japan.^[16–18]^

The code calls were widely available in acute-care hospitals, although small acute-care hospitals (23%) had no systematic protocols for hospital emergencies. From our search for articles, the prevalence of the code calls in the world worldwide was unknown; thus, we could not compare the rate to others. Interestingly, the public emergency call (119) was used in 13% of responding hospitals. Based on bed-function reports, approximately 58% of the total hospital beds were for acute-care in Japan in 2018. We selected defined acute-care hospitals (>75 beds); however, we did not consider the truthfulness of acute-care in medical treatments and procedures in eligible hospitals. Thus, studies that use the precise selection of hospital types could reveal the real need for response activity in hospital emergencies.

The respondents mainly opined on the requirements for improvement in the quantity and ability of staff. Koike et al^[15]^ reported that medical staff need more staff, knowledge, and education to manage clinical emergencies. However, the need for the involvement of medical safety management or data registration personnel was ranked low. DeVita et al^[24]^ published a statement of a consensus conference that the RRS comprises 4 components. They explained that evaluation systems that use data can improve patient safety if governed through a suitable structure. There is a gap between clinical needs and provider intention. As the new facilitating program is being introduced by the IHEC-J in Japan,^[25]^ further research could identify the interventions required to improve the activity of in-hospital emergencies.

There are several limitations to this study, mainly because of the nature of the questionnaire survey. The response rate of the survey is not sufficiently high to be extrapolated to a representative overview of Japanese acute-care hospitals. Several reasons for the low response rates were conceived. First, the department responsible for in-hospital patient emergency was not predetermined. Second, the survey was not an incentive because it was not related to regulation. Articles on questionnaires may be sophisticated for responders. Another limitation was that the survey did not include patient outcomes or real incidence of unexpected cardiac arrest in each hospital, which did not suggest that the RRS is required in smaller hospitals and shows a cost benefit.

## 5 Conclusion

The questionnaire survey revealed a lower prevalence of MET and RRT in smaller hospitals than in other hospitals. Respondents who experienced less satisfaction were keen to increase staff knowledge, supervisor presence, and a multidisciplinary approach, but merely to enrol data registration and involvement with the medical safety management sector. Further research is needed to clarify whether RRS in smaller hospitals shows efficacious patient outcomes or economic benefits.

## Acknowledgments

The authors thank all the study participants. They would like to thank Editage (www.editage.com) for English language editing.

## Author contributions

**Conceptualization:** Koji Hosokawa, Kohei Ota, Satoshi Yamaga, Junki Ishii, Nobuaki Shime.

**Data curation:** Koji Hosokawa, Hiroki Kamada.

**Formal analysis:** Koji Hosokawa, Hiroki Kamada, Junki Ishii.

**Investigation:** Koji Hosokawa, Hiroki Kamada, Junki Ishii, Nobuaki Shime.

**Methodology:** Koji Hosokawa, Hiroki Kamada.

**Project administration:** Koji Hosokawa, Kohei Ota, Nobuaki Shime.

**Resources:** Koji Hosokawa, Hiroki Kamada, Junki Ishii, Nobuaki Shime.

**Software:** Hiroki Kamada.

**Supervision:** Kohei Ota, Satoshi Yamaga, Nobuaki Shime.

**Validation:** Koji Hosokawa, Kohei Ota, Satoshi Yamaga, Junki Ishii, Nobuaki Shime.

**Visualization:** Koji Hosokawa.

**Writing – original draft:** Koji Hosokawa.

**Writing – review & editing:** Koji Hosokawa, Kohei Ota, Satoshi Yamaga, Junki Ishii, Nobuaki Shime.

## Supplementary Material

Supplemental Digital Content
