# Supplementary material for: Prevalence of rapid response systems in small hospitals: A questionnaire survey
Source: Medicine (Baltimore). 2021 Jun 11;100(23):e26261. doi: 10.1097/MD.0000000000026261 (PMC8202584; doi:10.1097/MD.0000000000026261)
Supplement: Supplemental Digital Content [file medi-100-e26261-s001.doc]

**Supplemental Table 1.** A list of questionnaires.

| **Question** | **Answer** |
| --- | --- |
| Department of the respondent | (Open-ended) |
| Does your hospital have rules or system for deteriorated patients or those who go into cardiac arrest? | Y/N |
| Does your hospital staff call 119 (public call for emergency service in fire bureau) when patients deteriorate or go into cardiac arrest? | Y/N |
| Does your hospital have a staff call for non-specified staff in an emergency? | Y/N |
| Is this present 24/7? | Y/N |
| Does your hospital have an emergency call for preassigned response doctors in an emergency? | Y/N |
| Is this available 24/7? | Y/N |
| Does your hospital have a MET call? | Y/N |
| Is this available 24/7? | Y/N |
| Does your hospital have other facilities when patients deteriorate or go into cardiac arrest? If yes, what are they? | Y/N, (open-ended) |
| Does your hospital have rules or systems that are activated before patients obviously deteriorate? | Y/N |
| Does your hospital have an RRT call for patients who are predicted to deteriorate? | Y/N |
| Is this available 24/7? | Y/N |
| Does your hospital have a CCOT round? | Y/N |
| Is this available 24/7? | Y/N |
| Does your hospital have other facilities for patients who are predicted to deteriorate? If yes, what are they? | Y/N, (open-ended) |
| Which areas do you think have a need for improvement of response to patient emergency with regard to resources? (multiple answers allowed) | Improvement of RRS; Increased number of bedside monitors; Increased number of intensive care beds; Others |
| Which do you think are areas for improvement of response to patient emergency with regard to staff? (multiple answers allowed) | Knowledge of emergency; Presence of supervisors; Positive attitude of staff; Number of staff; Smooth communication among departments; Others |
| Which do you think are areas for improvement of response to patient emergency with regard to the system or rules of the hospital? (multiple answers allowed) | Review and feedback; Multidisciplinary team approach; Involvement of medical safety management; Others |
| Which do you think are areas for improvement in response to patient emergency with regard to social factors? (multiple answers allowed) | Increase in payment from insurance provider; Submission to a registry; Report to public sectors; Others |
| Does your hospital collect data of the recent 1 year? | Y/N |
| Does your hospital register data in the IHEC-J registry? | Y/N |
| How many (minimum number) anaesthesiologists work during the day? | (number only) |
| How many (minimum number) emergency doctors work during the day? | (number only) |

Table legends:

The survey questionnaire was developed in Google Forms^TM^ and included articles above. CCOT, critical care outreach team; MET, medical emergency team; N, no; RRT, rapid response team; Y, yes.
